# ZAP-X Stereotactic Radiosurgery: The Initial Experience Involving 200 Patients

**DOI:** 10.7759/cureus.98320

**Published:** 2025-12-02

**Authors:** Timothy Chen, Patrick Pema, Michael Chaga, Wenzheng Feng, Tingyu Wang, Darra Conti, Jing Feng, Akil Anthony, Ma Rhudelyn Rodrigo, Harshal Shah, Daniel Monahan, Elizabeth Luick, Daniel Thompson, Joy Baldwin, Brielle Latif, Georgia Montone, Joseph Hanley, Nitesh V Patel, Shabbar Danish

**Affiliations:** 1 Radiation Oncology, Jersey Shore University Medical Center, Neptune, USA; 2 Neurosurgery, Hackensack Meridian School of Medicine, Hackensack, USA; 3 Genetics and Statistics, Rutgers University, New Brunswick, USA; 4 Neurosurgery, Jersey Shore University Medical Center, Neptune, USA

**Keywords:** gyroscopic, radiosurgery, srs, stereotactic radiosurgery, zap-x

## Abstract

The ZAP-X (ZAP Surgical Systems, Inc., San Carlos, CA) is the newest dedicated cranial stereotactic radiosurgery (SRS) platform. A prospective study was conducted between October 2023 and January 2025 involving 200 patients with 374 intracranial targets treated using ZAP-X. The study aimed to assess the feasibility of the ZAP-X workflow, as well as its safety, clinical and dosimetric performance, and patient satisfaction. The study cohort included various central nervous system pathologies, including 106 brain metastases (BM), 42 meningiomas (MA), 18 trigeminal neuralgias (TN), 11 acoustic neuromas (AN), nine recurrent glioblastomas (RGBM), seven pituitary adenomas (PA), three spinal tumors (ST), three arteriovenous malformations (AVM), and one cavernous hemangioma of the pons (HM).

The average number of patients treated per month was 13 ± 2, and the average number of treatments per month was 35 ± 9. The maximum number of patients treated in a day was five. The average gamma passing rate was 98.5 ± 1.7% (91.2 - 100%) at a 10% low-dose threshold, 2% dose difference, and 1 mm distance-to-agreement. The average conformity index (CI), Paddick conformity index (PCI), gradient index (GI), homogeneity Index (HI), and gradient measure across all patients were 1.3 ± 0.3, 0.77 ± 0.11, 3.3 ± 0.8, 1.5 ± 0.3, and 0.38 ± 0.16 cm, respectively. The largest tumor volume treated was 29.29 cm^3,^ and the highest number of targets treated in a single course was 10. Average treatment time was 50 ± 20 minutes, with setup time being the most significant predictor (adjusted r² = 0.972). The Efficast head and shoulder mask significantly improved patient comfort and reduced alignment errors compared to head-only masks. Patient satisfaction was high across all measured domains, with average scores ranging from 4.7 to 5.0 on a 5-point scale. The most common treatment side effects were fatigue and headache.

This initial experience represents the largest single institution series to date and supports the ZAP-X as a promising alternative to conventional SRS platforms such as Gamma Knife (GK) and CyberKnife (CK). Continued research and multi-institutional studies are warranted to further validate these findings and assess long-term outcomes.

## Introduction

The ZAP-X (ZAP Surgical Systems, Inc., San Carlos, CA) is the newest dedicated cranial stereotactic radiosurgery (SRS) platform. The radiation source for the ZAP-X is a 3 MV S-band linear accelerator (Linac) mounted on axial and oblique gimbals, which allows for isocentric, non-coplanar, dual-axis radiation delivery over a solid angle of greater than 2π steradians. The flattening filter free beam is collimated via a rotating tungsten wheel with eight circular field sizes ranging from 4 to 25 mm in diameter, defined at a source-to-axis distance (SAD) of 45 cm. The self-shielded ZAP-X system design consists of a shielded treatment chamber, rotating shell, and a pneumatic door located at the foot of the table. This design reduces radiation exposure inside the treatment room, eliminating the need for a shielded vault and increasing installation flexibility [1-3]. The ZAP-X produced the lowest full-width half maximum and sharpest overall penumbra for its smallest collimator compared to beam profile parameters of Gamma Knife (GK) and CyberKnife (CK) for the corresponding smallest collimator [4].

The objective of this study is to evaluate ZAP-X workflow feasibility, safety, clinical and dosimetric performance, and patient satisfaction. Analysis of this newly pioneered system is important to gauge its efficacy and refine its specificity for varying patients and conditions. Our report on the first 200 patients treated with the ZAP-X SRS platform is particularly significant given the novelty of the technology and the current scarcity of extensive literature on it [5-8]. This review provides first-hand, valuable insights into dosimetric characteristics, clinical efficacy, pathology-specific treatment parameters, and patient satisfaction. To date, it represents the largest single-institution series treated with the ZAP-X platform.

## Materials and methods

The data were collected prospectively on patients receiving ZAP-X SRS who were treated between October 2023 and January 2025 at Jersey Shore University Medical Center (JSUMC). The study protocol was reviewed and approved by the Institutional Review Board, Pro2024-0029. The patient cohort comprised individuals who met clinical indications for treatment with ZAP-X SRS.

Clinical workflow

**Figure 1:**
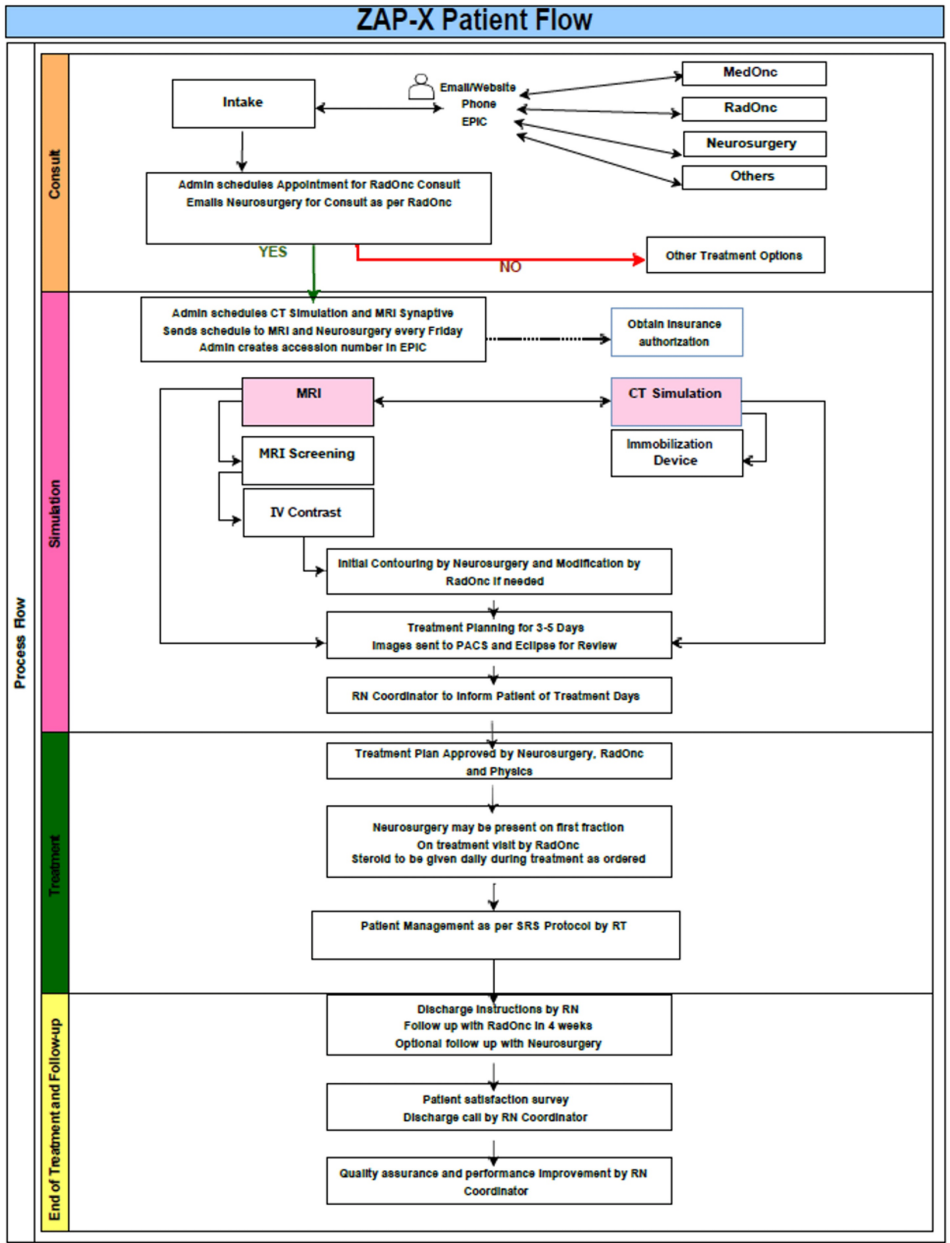
ZAP-X clinical workflow

Our workflow process consists of four stages: consultation, simulation, treatment, and end of treatment and follow-up. At the beginning of the simulation phase, CT imaging is used to create an immobilization mask, and on-site planning MRI is performed, and imaging is sent to Eclipse for fusion and contouring. Imaging data is then processed and sent to PACS and the Eclipse (Varian Medical Systems, Palo Alto, CA, version 15.6) treatment planning system (TPS) for contouring and treatment planning. The treatment phase begins with treatment plan approval by the neurosurgeon, radiation oncologist, and physicist. The neurosurgeon and radiation oncologist are required to be present for the first treatment fraction. The last phase - end of treatment and follow-up - begins with patient discharge per the registered nurse's instruction. Follow-up with both radiation oncology and neurosurgery occurs four weeks post-SRS. A patient satisfaction survey utilizing a 5-point Likert scale is completed by each patient. Lastly, quality assurance (QA) and performance improvement are handled by the nurse coordinator. A diagram of the ZAP-X clinical workflow is shown in Figure 1.

All patients underwent planning MRI with or without gadolinium contrast on a 0.5 T Synaptive MRI co-located in a room adjacent to the ZAP-X as described by Chaga et al. [9]. Patients were scheduled for same-day CT Sim and MRI with a goal of ≤5 days between consult and imaging for benign cases and same-day consult and imaging for malignant cases. After imaging, patients were scheduled for SRS treatment with a goal of ≤5 days between imaging and treatment. Volumes of interest were contoured by a qualified radiation oncologist, neurosurgeon, and medical physicist utilizing a fused dataset comprising treatment planning CT and post-gadolinium MRI within the Eclipse TPS. The contoured images were then imported to the ZAP-X TPS (versions 1.8 - 1.10).

Treatment planning was performed using either forward-planning or combined forward- and inverse-planning modes. In forward-planning mode, isocenters were manually placed in the target by the planner. In combined forward- and inverse-planning mode, isocenters were manually placed first, followed by inverse optimization of beam weights and manual addition, deletion, or repositioning of isocenters. A 0.5 mm dose grid was utilized, limiting the dose to the eyes, lens, optic nerves, optic chiasm, cochleae, brainstem, and spinal cord based on Timmerman organ-at-risk (OAR) recommendations [10]. Plan quality was evaluated for conformality, dose fall-off, and dose homogeneity using conformity index (CI), Paddick conformity index (PCI), gradient index (GI), homogeneity index (HI), and gradient measure (GM).

The CI was defined as the ratio of the prescription isodose volume to the target volume [11]. The PCI was calculated as the square of the target volume covered by the prescription isodose divided by the product of the target volume and the prescription isodose volume [12]. The GI was defined as the ratio of the volume receiving half the prescription dose to the prescription isodose volume [13]. The HI was calculated as the ratio of the maximum dose to the prescription dose [14]. The GM was defined as the difference, in centimeters, between the equivalent sphere radii of the 50% and 100% prescription isodose line volumes [15]. Plan quality constraints followed the Radiation Therapy Oncology Group Protocol 0813 [16].

Patient-specific and machine quality assurance

For each patient case, a patient-specific quality assurance (PSQA) program was utilized to ensure high accuracy of the planned versus delivered dose. PSQA consisted of two components: independent dose calculation and absolute planar dose measurements. For independent dose calculation, a software developed in-house called ZapMU was utilized [17-19]. For absolute planar dose measurements, SRS MapCHECK was utilized along with SNC Patient software (Sun Nuclear, Melbourne, FL) for gamma analysis [20-21]. Machine daily QA is performed once each day before each treatment, along with monthly and annual QA. All ZAP-X QA tests are summarized in Table 1.

**Table 1:** JSUMC ZAP-X daily, monthly, and annual QA procedures with setup configuration and equipment SAD: source-to-axis distance; MU: monitor units; TPS: treatment planning system; SSD: source-to-axis distance; D10cm: dose at 10 cm depth; D0.7cm: dose at 0.7 cm depth; 0°: North Pole gantry angle; PDD: percent depth dose

Procedure	Tolerance
Daily	
Safety/imaging	
Software, hardware, and exclusion zone E-Stop	Functional
Table, shell, and door	Initialized, move to intended positions
Gantry rotation	Initialized, move to intended positions
Proximity error check	Interlock tripped when close to the table
Beam on indicators	Functional
Audio/visual	Functional
Pendant	Functional, E-Stop functional
kV imaging (accuracy of table and gantry position)	Baseline
MV imaging (consistency of beam positioning and output)	Baseline
Winston-Lutz (Hayes Phantom, collimator = 15 mm, 6 gantry angles)	
Translational target and beam offsets	<1 mm
Output check (PTW Semiflex 31021, collimator = 25 mm, 0°, 500 MU, 45 cm SAD)	
Absolute dose output in air	± 2% baseline
Primary and secondary MU difference	≤3%
Monthly	
Safety	
Emergency button	Functional
Door release	Functional
Shell release	Functional
kV imaging (Leeds Phantom, Gammex Tool)	
kV homogeneity, kV high contrast, low contrast	± 5% baseline
MV imaging (all collimators, 0°, 200 MU)	
MV cone center and size consistency	<1 mm
End-to-end (Hayes Phantom, EBT3 film, collimator = 25 mm, 45 cm SAD)	
Orthogonal film difference	<1 mm
End-to-end (SRS MapCHECK, gamma criteria 10%/2%/1 mm)	≥95%
TPS integrity and plan consistency	<1%
Output check (PTW Semiflex 31010, Sphere Phantom, collimator = 25 mm, 0°, 200 MU, 50 cm SAD)	
Absolute dose output	± 2% baseline
Energy check (PTW Semiflex 31010, Column Phantom, collimator = 25 mm, 0°, 200 MU, 45 cm SSD)	
D10cm/D0.7cm	± 2% baseline
Daily QA	Refer to daily
Annually	
Absolute output (PTW Semiflex 31010, PTW MP3-XS, collimator = 25 mm, 0°, 500 MU, 45 cm SSD, 5 cm depth)	
Dmax at 45 cm SAD	± 2% nominal
Output factor comparison with TPS (MicroSilicon, all collimators, 0°, 45 cm SAD)	
Output factor at Dmax normalized with a 25 mm collimator	± 2% commissioning
PDD comparison (MicroSilicon, PTW MP3-XS, 45 cm SSD)	
Measured and TPS PDD difference	± 1% commissioning
Profile comparison (MicroSilicon, PTW MP3-XS, 45 cm SSD)	
Measured and TPS profile difference	0.1 mm or ± 1% commissioning
Start shot test (Star shot fixture, EBT3 film, collimator = 5 mm, 5 gantry angles, 500 MU, 45 cm SAD)	
Radius	± 1 mm baseline
Chamber cross calibration (PTW Semiflex 31021, collimator = 25 mm, 0°, 500 MU, 45 cm SAD)	
Charge ratio of calibrated to cross-calibrated chambers	± 1% baseline
External audit with RDS (cylindrical phantom, TLD, 45 cm SAD, 7 mm depth)	
Measured and RDS absolute dose difference	± 5%
PSQA with timer and interrupt test (SRS MapCHECK, gamma criteria 10%/2%/1 mm)	
Simulation plan delivery to the chamber isocenter	≥95%
Monthly and daily QA	Refer to monthly and daily

ZAP-X thermoplastic masks

**Figure 2:**
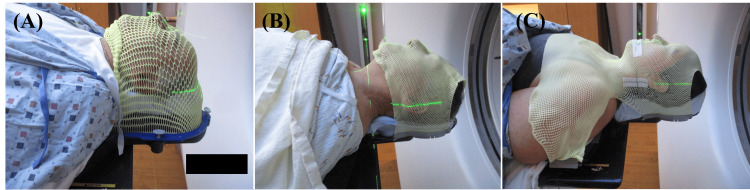
ZAP-X thermoplastic masks utilized at JSUMC (A) Fibreplast head mask, (B) Nanor head mask. (C) Efficast head and shoulder mask with two shims JSUMC: Jersey Shore University Medical Center

To ensure patient comfort and precise positioning, patients were placed in mold care and thermoplastic Fibreplast head masks (CQ Medical, Avondale, PA) [22], Nanor head masks (Orfit Industries, Jericho, NY) [23], and Efficast head and shoulder masks (Orfit Industries, Jericho, NY) [24]. All masks are shown in Figure 2.

For the Efficast masks, two shims were employed during mask creation. For mask comparison, patient comfort, number of readjustments, setup time, and alignment offsets were evaluated for 60 patients: 30 Fibreplast and Nanor head mask patients and 30 Efficast mask patients. The patient alignment process involves a sequence of 3D alignment steps using non-coaxial kV X-ray images from multiple gantry angles. The images are co-registered to digitally reconstructed radiographs (DRRs) generated from the initial CT used in the treatment plan. The initial auto-alignment before treatment was approved by a radiation oncologist. Alignment deviation of more than 2 mm in any direction and 1.5° in any rotation axis would require a readjustment of the patient positioning and repeating auto-alignment and radiation oncologist approval. If >5 gantry locations with MV dosimetry deviation of more than 10% exist, a physicist's investigation is required. Treatment would be stopped for further investigation with the vendor if >10 gantry locations with MV dosimetry deviation of more than 10% exist. Patient alignment offsets and the number of readjustments were obtained from system log files on the treatment console. Mask comfort was evaluated using a Likert scale from 1-5 (1 = “very poor”, 5 = “very good”).

Patient satisfaction

Patient satisfaction was measured using a postoperative questionnaire, which utilized a 5-point Likert scale. Patients were asked to rate their experience in the following categories: ease of scheduling, staff helpfulness, ZAP-X cleanliness, staff friendliness/courtesy, physician procedure explanation, comfort of waiting area, comfort during simulation, comfort during mask creation, comfort during MRI, comfort during treatment, comfort post-treatment, response to concerns/complaints, overall rating of care, care coordination, and recommending JSUMC.

ZAP-X treatment time

As a clinical endeavor to reduce the total treatment time, the treatment time variables for each patient were obtained from reports generated by the ZAP-X system upon completion of treatment. Setup time was defined as the duration required for auto-alignment imaging and its subsequent review. Gantry time was the duration for the gantry to move all gantry angles throughout the entire delivery. Table time was the duration for the treatment table to move to all table locations throughout the entire delivery. kV imaging and processing time was the duration to acquire the kV images throughout the entire delivery, match to DRRs, and perform noise reduction, edge detection, and normalization. Linac time was the beam-on delivery duration. Treatment time was the duration from initial auto-alignment to the end of treatment. Calculated treatment time was the predicted treatment duration by the ZAP-X TPS.

Statistical analysis

Statistical analysis was conducted in Stata 18 (Release 18, StataCorp LLC, College Station, TX) and R version 4.4.1, utilizing mean, standard deviation (SD), median, interquartile range (IQR), range, and linear regression. Correlations among the various dosimetric parameters and indices were examined using Pearson correlation coefficients r and 95% confidence interval (95% CI) using Fisher’s transforms. Variables were considered correlated if |r| > √0.6 and 95%CI > 0. Two-sample t-test with Welch correction was performed for mask comfort comparison. Two-sample Wilcoxon rank-sum (Mann-Whitney) test was performed for the mask number of readjustments, setup time, and offset comparisons. Statistical significance was defined as p<0.05. ZAP-X treatment time was modeled using multivariate linear regression to determine variable importance. Clinical outcome was assessed using Kaplan-Meier survival analysis.

## Results

A total of 200 patients were treated with ZAP-X SRS from October 2023 to January 2025, consisting of 374 targets. Patient pathology demographics consisted of 106 brain metastases (BM), 42 meningiomas (MA), 18 trigeminal neuralgias (TN), 11 acoustic neuromas (AN), nine recurrent glioblastomas (RGBM), seven pituitary adenomas (PA), three spinal tumors (ST), three arteriovenous malformations (AVM), and one cavernous hemangioma of the pons (HM). The average number of patients treated per month was 13 ± 2, and the average number of treatments per month was 35 ± 9.

The maximum number of patients treated in a day was five. Self-reported physicist treatment planning time was as follows: TN: ≤5 minutes, single target spherical: ≤30 minutes, multiple targets spherical: ≤1 hour, irregular single target or multiple targets = minutes - three days, PSQA = hours. From October 2023 to January 2025, five patients were postponed due to machine issues, with no cancellations. All QA tests and measurements were within the recommended tolerances. The average Winston-Lutz x-offset was 0.04 ± 0.13 mm (range: -0.32 - 0.36 mm), the average y-offset was 0.38 ± 0.09 mm (range: -0.15 - 0.58 mm), and the average z-offset was 0.1 ± 0.1 mm (range: -0.13 - 0.66 mm). The average PSQA gamma passing rate was 98.5 ± 1.7% (range: 91.2 - 100%) at a 10% low-dose threshold, 2% dose difference, and 1 mm distance-to-agreement.

Dosimetric evaluation

Various dosimetric variables for each pathology are presented in Table 2. The average delivered doses were 23 ± 4 Gy for BM, 23 ± 4 Gy for MA, 83 ± 5 Gy for TN, 21 ± 5 Gy for AN, 30 Gy for RGBM, 26 ± 3 Gy for PA, 34 ± 6 Gy for AVM, 28 ± 3 Gy for ST, and 25 Gy for HM. The largest tumor volume treated was 29.29 cm³, and the maximum number of targets treated in a single course was 10. Regarding plan quality, the average indices were as follows: CI: 1.3 ± 0.3, PCI: 0.77 ± 0.11, GI: 3.3 ± 0.8, HI: 1.5 ± 0.3, and GM: 0.38 ± 0.16. Mean target prescription dose coverage was 98 ± 2%. Strong correlations were found between the following dosimetric variables: monitor units and Linac time (r = 0.995, 95%CI = 0.994 - 0.996), GM and target volume (r = 0.888, 95% CI = 0.864 - 0.908), treatment time and setup time (r = 0.880, 95% CI = 0.860 - 0.898), number of beams and calculated treatment time (r = 0.870, 95% CI = 0.848 - 0.889), HI and prescription isodose line (r = -0.846, 95% CI = -0.873 - -0.814), dose and number of fractions (r = 0.832, 95% CI = 0.804 - 0.856), number of isocenters and calculated treatment time (r = 0.816, 95% CI = 0.786 - 0.842), CI and PCI (r = -0.814, 95% CI = -0.846 - -0.777), total collimator size and number of isocenters (r = 0.813, 95% CI = 0.783 - 0.839), treatment time and gantry time (r = 0.800, 95% CI = 0.768 - 0.828), and total path number and number of isocenters (r = 0.783, 95% CI = 0.748 - 0.813).

**Table 2:** Dosimetric variables for each pathology Values presented as average ± standard deviation and range BM: brain metastases; MA: meningiomas; TN: trigeminal neuralgias; AN: acoustic neuromas; RGBM: recurrent glioblastomas; PA: pituitary adenomas; ST: spinal tumors; AVM: arteriovenous malformation; HM: cavernous hemangioma of pons; CI: conformity index; PCI: Paddick conformity index; GI: gradient index; HI: homogeneity index; GM: gradient measure; -: not applicable

	BM (106)	MA (42)	TN (18)	AN (11)	RGBM (9)	PA (7)	AVM (3)	ST (3)	HM (1)	All (200)
Rx dose (Gy)	23 ± 4	23 ± 4	83 ± 5	21 ± 5	30	26 ± 3	34 ± 6	28 ± 3	25	29 ± 17
	18 – 30	16 – 30	80 – 90	14 – 30		21 – 30	27 –37.5	24 – 30		14 – 90
Rx isodose line (%)	60 ± 10	59 ± 5	100	59 ± 6	57 ± 8	62 ± 7	61 ± 5	62 ± 5	57	66 ± 14
	50 – 89	52 – 75		51 – 71	50 – 75	53 – 70	55 – 64	56 – 66		50 – 100
Number of targets	3 ± 2	1.1 ± 0.4	1	1.2 ± 0.4	1.2 ± 0.7	1	1	1	1	1.9 ± 1.7
	1 – 10	1 – 3		1 – 2	1 – 3					1 – 10
Target volume (cm^3^)	3 ± 5	6 ± 6	0.05 ± 0.03	3 ± 2	10 ± 4	8 ± 9	9 ± 4	3 ± 3	1.9	4 ± 6
	0.05 – 29.29	0.27 – 24.57	0.01 – 0.11	0.12 – 8.33	3.66 – 19.36	0.79 – 26.4	4.84 –11.56	0.61 – 6.29		0.01 – 29.29
Number of fractions	2.5 ± 1.6	4 ± 4	1	3.2 ± 1.1	5	4 ± 1	4.3 ± 1.2	4.3 ± 1.2	5	2.8 ± 1.6
	1 – 5	1 – 5		1 – 5		3 – 5	3 – 5	3 – 5		1 – 5
Number of isocenters	7 ± 4	10 ± 3	1	8 ± 4	10 ± 3	12 ± 4	10 ± 3	12 ± 4	12	8 ± 4
	1 – 18	4 – 15		1 – 16	6 – 15	6 – 17	8 – 13	8 – 16		1 – 18
Number of beams	180 ± 80	190 ± 50	200 ± 70	180 ± 50	240 ± 50	200 ± 60	260 ± 70	280 ± 70	266	190 ± 70
	31 – 429	74 – 289	130 – 330	68 – 233	162 – 325	122 – 321	177 –318	212 – 257		31 – 429
Target Rx coverage (%)	99 ± 2	98 ± 1	-	97.8 ± 1.2	98.0 ± 1.2	98.8 ± 1.1	97.6 ± 0.6	97 ± 1	99.36	98 ± 2
	71.28 – 100	95.91 – 99.80		95.47 – 99.73	95.43 – 99.42	96.44 – 99.82	97.22 – 98.33	96.19 – 98.16		71.28 – 100
CI	1.3 ± 0.3	1.26 ± 0.17	-	1.2 ± 0.2	1.2 ± 0.1	1.28 ± 0.08	1.22 ± 0.04	1.13 ± 0.06	1.17	1.3 ± 0.3
	0.71 – 3.87	1.08 – 1.92		1.06 – 1.86	1.04 – 1.41	1.18 – 1.37	1.18 – 1.26	1.07 – 1.17		0.71 – 3.87
PCI	0.76 ± 0.12	0.78 ± 0.08	-	0.79 ± 0.09	0.85 ± 0.11	0.76 ± 0.04	0.78 ± 0.03	0.84 ± 0.03	0.85	0.77 ± 0.11
	0.26 – 1.33	0.51 – 0.89		0.54 – 0.81	0.70 – 1.12	0.72 – 0.84	0.75 – 0.81	0.81 – 0.87		0.26 – 1.33
GI	3.4 ± 0.8	2.9 ± 0.5	-	3.0 ± 0.3	2.9 ± 0.7	2.78 ± 0.14	2.9 ± 0.2	3.0 ± 0.5	2.60	3.3 ± 0.8
	2.13 – 7.77	2.33 – 5.45		2.60 – 3.68	2.34 – 4.93	2.59 – 3.03	2.68 – 3.14	2.58 – 3.54		2.13 – 7.77
HI	1.5 ± 0.2	1.69 ± 0.16	-	1.70 ± 0.17	1.8 ± 0.2	1.63 ± 0.18	1.66 ± 0.14	1.63 ± 0.14	2.60	1.5 ± 0.3
	1.08 – 2.02	1.31 – 1.92		1.41 – 1.96	1.33 – 2	1.43 – 1.89	1.56 – 1.82	1.52 – 1.79		1.08 – 2.60
GM (cm)	0.35 ± 0.16	0.44 ± 0.14	-	0.38 ± 0.11	0.6 ± 0.1	0.50 ± 0.17	0.57 ± 0.04	0.38 ± 0.08	0.30	0.38 ± 0.16
	0.13 – 1.06	0.20 – 0.79		0.19 – 0.65	0.48 – 0.76	0.28 – 0.73	0.53 – 0.61	0.29 – 0.43		0.13 – 1.06
Treatment time (min/Fx)	50 ± 20	49 ± 16	35 ± 7	48 ± 19	66 ± 19	40 ± 10	65 ± 11	58 ± 9	40 ± 1.1	50 ± 20
	9 – 141	25 – 133	28 – 52	12 – 84	38 – 118	29 – 66	42 – 84	44 – 73	39 – 42	9 – 141

Mask evaluation

Mask parameter results for the Fibreplast and Nanor head mask, and the Efficast head and shoulder mask, are shown in Table 3. The shim mask significantly reduced y-offset (p = 0.0014), pitch-offset (p<0.001), roll-offset (p<0.001), yaw-offset (p<0.001), number of readjustments (p = 0.0294), and setup time (p = 0.0017). There was no significant difference in x-offset (p = 0.406) and z-offset (p = 0.453). Mask comfort improved significantly (p = 0.0345).

**Table 3:** Mask parameter results for Fibreplast and Nanor head mask and Efficast head and shoulder mask ^†^Median (IQR: p25, p75). ^‡^P<0.05 indicates statistical significance Two-sample t-test with Welch correction was performed for mask comfort comparison. Two-sample Wilcoxon rank-sum (Mann-Whitney) test was performed for readjustments, setup time, and offset comparisons SD: standard deviation; IQR: interquartile range

Parameter	Mask	Average ± SD	Median^†^	Range	P=value^‡^
Comfort	Head (n = 30)	4.1 ± 1.0	4 (3.5, 5)	2 – 5	0.0345
	Efficast (n = 30)	4.7 ± 0.7	5 (4.5, 5)	3 – 5	
Readjustments	Head (n = 30)	5 ± 4	4 (1, 7)	0 – 13	0.0194
	Efficast (n = 30)	2 ± 3	1 (0, 4)	0 – 12	
Setup time (min)	Head (n = 30)	17 ± 11	15 (7, 24)	1 – 47	<0.001
	Efficast (n = 30)	10 ± 10	4 (2, 11)	1 – 51	
x-offset (mm)	Head (n = 30)	0.0 ± 0.4	0 (-0.15, 0.14)	-3.94 – 4.31	0.406
	Efficast (n = 30)	0.0 ± 0.3	0 (-0.12, 0.11)	-2.76 – 2.48	
y-offset (mm)	Head (n = 30)	0.0 ± 0.3	0 (-0.11, 0.11)	-6.57 – 2.28	0.0014
	Efficast (n = 30)	0.0 ± 0.3	0 (-0.08, 0.11)	-3.06 – 3.61	
z-offset (mm)	Head (n = 30)	0.0 ± 0.3	0 (-0.12, 0.14)	-3.12 – 6.53	0.453
	Efficast (n = 30)	0.0 ± 0.3	0 (-0.11, 0.12)	-3.01 – 2.22	
Pitch-offset (°)	Head (n = 30)	0.1 ± 0.8	0.15 (-0.61, 0.79)	-2.79 – 2.04	<0.001
	Efficast (n = 30)	0.0 ± 0.5	-0.09 (-0.46, 0.72)	-2.64 – 2.01	
Roll-offset (°)	Head (n = 30)	-0.2 ± 0.8	-0.20 (-0.87, 0.36)	-3.05 – 2.40	<0.001
	Efficast (n = 30)	0.0 ± 0.6	-0.16 (-0.72, 0.26)	-1.87 – 1.68	
Yaw-offset (°)	Head (n = 30)	0.0 ± 0.8	-0.22 (-0.62, 0.62)	-2.18 – 2.71	<0.001
	Efficast (n = 30)	0.0 ± 0.8	-0.12 (-0.52, 0.50)	-2.11 – 2.08	

Patient satisfaction

100 patients responded to the postoperative satisfaction survey. The average patient ratings were as follows: ease of scheduling: 4.9 ± 0.3, staff helpfulness: 4.98 ± 0.13, ZAP-X cleanliness: 4.97 ± 0.18, staff friendliness/courtesy: 4.98 ± 0.13, physician procedure explanation: 4.9 ± 0.3, waiting area comfort: 4.9 ± 0.3, simulation comfort: 4.7 ± 0.6, mask creation comfort: 4.6 ± 0.6, MRI comfort: 4.7 ± 0.6, treatment comfort: 4.4 ± 0.9, post-treatment comfort: 4.7 ± 0.5, response to concerns/complaints: 4.9 ± 0.3, overall rating of care: 4.97 ± 0.18, care coordination: 5.0 ± 0.2, and recommending JSUMC: 4.98 ± 0.13.

ZAP-X treatment time

**Figure 3:**
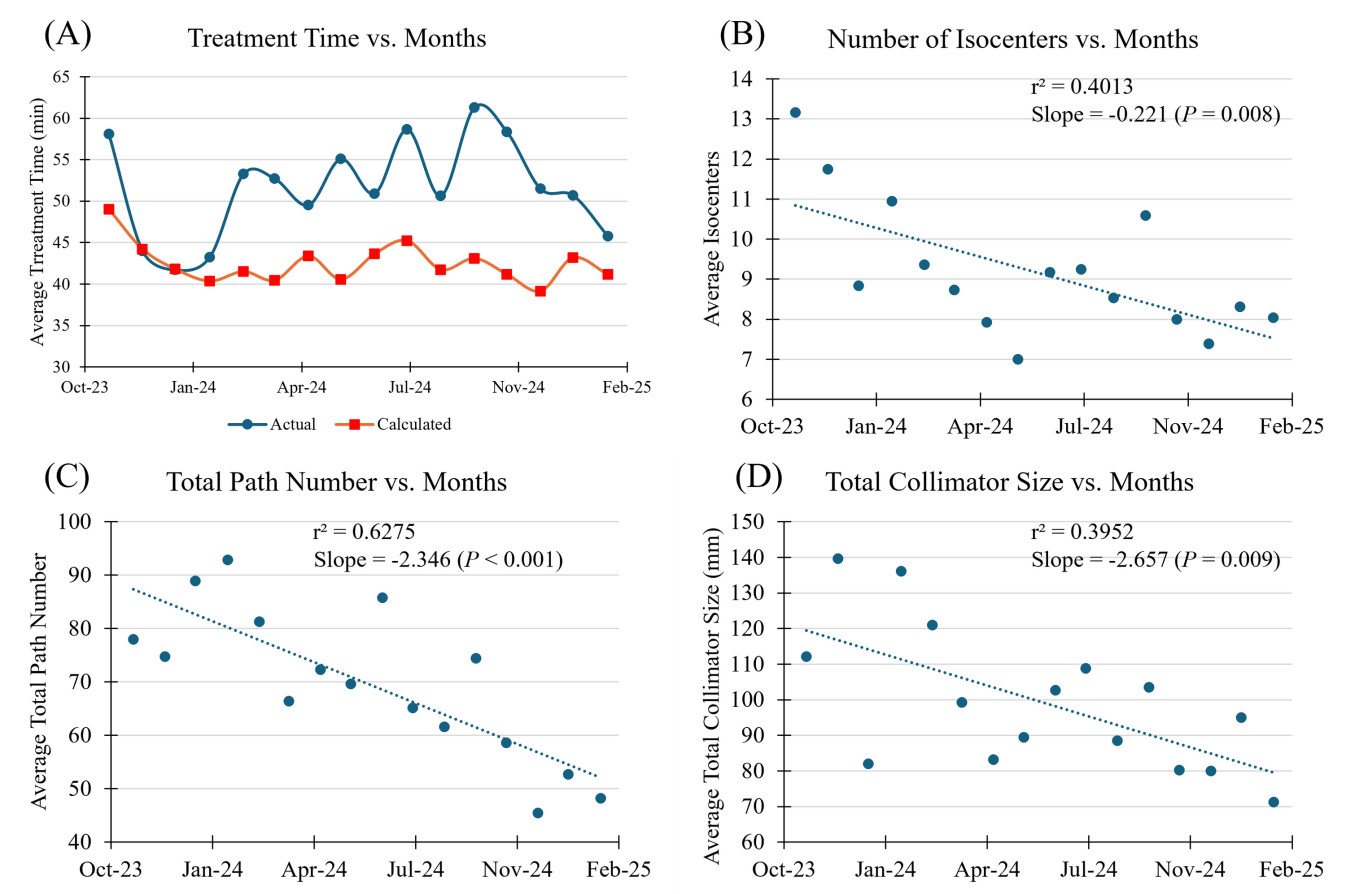
(A) Treatment time, (B) number of isocenters, (C) total path number, and (D) total collimator size over months The treatment time appears oscillatory in nature, increasing and decreasing periodically as the months increase. Calculated treatment time appears higher in the first month and levels out in the second month. The higher calculated treatment time in the beginning may be due to a learning curve in treatment planning

ZAP-X treatment time variables are presented in Table 4. The average treatment time was 50 ± 20 minutes, calculated treatment time 40 ± 10 minutes, setup time 11 ± 12 minutes, gantry time 30 ± 10 minutes, table time 0.8 ± 0.5 minutes, kV imaging and processing time 8 ± 4 minutes, and Linac time 6 ± 3 minutes. Average treatment time and calculated treatment time plotted over months are shown in Figure 3A. The treatment time appears oscillatory in nature, increasing and decreasing periodically as the months increase. Calculated treatment time appears higher in the first month and levels out in the second month. The higher calculated treatment time in the beginning may be due to a learning curve in treatment planning, supported by plotting over months the number of isocenters (slope = -0.221, p = 0.008), total path number (slope = -2.657, p = 0.009), and total collimator size (slope = -2.346, p<0.001), as shown in Figures 3B-3D.

**Table 4:** JSUMC ZAP-X treatment time variables ^†^Median (IQR: p25, p75) Setup time refers to the duration required for auto-alignment imaging and image review. Gantry time represents the total time needed for the gantry to move through all planned angles during delivery. Table time corresponds to the time taken for the treatment table to move to each required position. kV imaging and processing time includes acquiring kV images throughout delivery and performing DRR matching, noise reduction, edge detection, and normalization. Linac time denotes the beam-on portion of the treatment IQR: interquartile range; DRR: digitally reconstructed radiograph

	Average	Median^†^	Range
Treatment time (min/Fx)	50 ± 20	49 (39, 63)	9 – 141
Calculated treatment time (min/Fx)	40 ± 10	43 (37, 48)	16 – 72
Setup time (min/Fx)	11 ± 12	7 (4, 15)	1 – 81
Gantry time (min/Fx)	30 ± 10	25 (19, 31)	2 – 73
Table time (min/Fx)	0.8 ± 0.5	0.73 (0.55, 0.98)	0.05 – 6
kV imaging and processing time (min/Fx)	8 ± 4	7.58 (4.975, 10.28)	0.1 – 26
Linac time (min/Fx)	6 ± 3	5 (4, 7)	1 – 23

**Figure 4:**
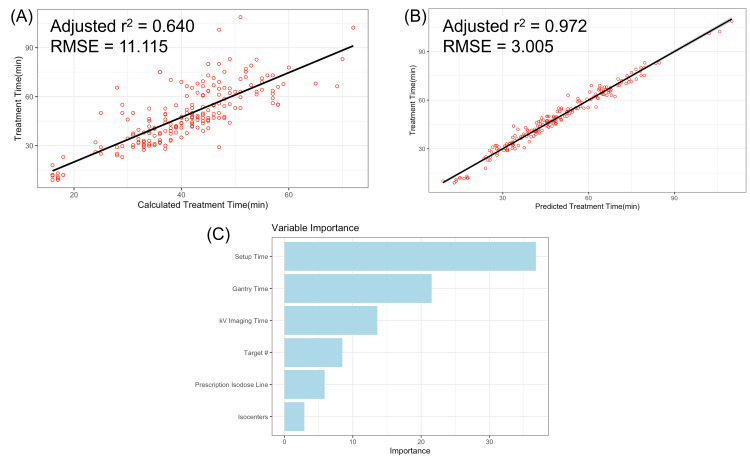
(A) Treatment time vs. calculated treatment time. (B) A multivariate linear regression was conducted with six variables: setup time, gantry time, kV imaging and processing time, number of targets, prescription isodose line, and number of isocenters. (C) Variable importance graph: setup time had the greatest effect on treatment duration RMSE: root mean square error

The number of isocenters, total path number, and total collimator size used by the physicist treatment planners decreased significantly over time. To assess the accuracy of the ZAP-X TPS calculated treatment time, linear regression was performed against treatment time, producing an adjusted r^2^ of 0.640 with a root mean square error (RMSE) of 11.115, as shown in Figure 4A. The ZAP-X TPS model’s predictions for treatment time deviate on average by 11 minutes from the actual treatment durations. For the multivariate model of treatment time, three multivariate models were analyzed. The model utilizing setup time, gantry time, kV imaging and processing time, number of targets, number of isocenters, and prescription isodose line had residuals that were the most normally distributed. Additionally, the model achieved an adjusted r² of 0.972, indicating that it captures almost all variability in treatment time, and with an RMSE of 3.005, the model’s predictions on average deviate by three minutes from the actual treatment durations. The regression plot for the multivariate model is shown in Figure 4B. In this model, all predictors were statistically significant. The model revealed that setup time was the most important predictor of treatment duration, followed by gantry time and kV imaging and processing time. The model also demonstrated that the number of targets, the prescription isodose line, and the number of isocenters significantly impacted treatment time. The variable importance graph for the multivariate model is shown in Figure 4C.

Treatment side effects

**Figure 5:**
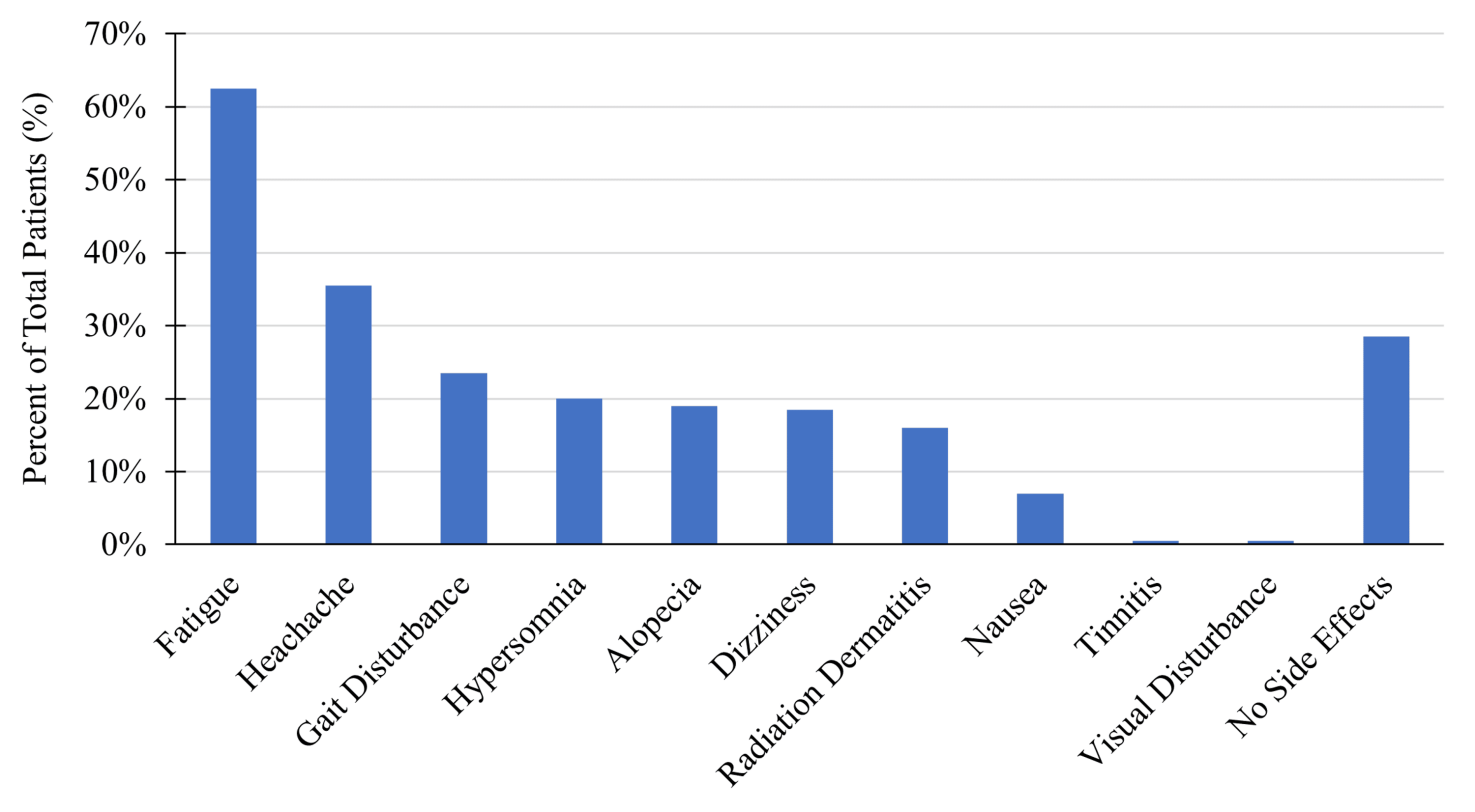
ZAP-X SRS treatment side effects SRS: stereotactic radiosurgery

Treatment side effects observed after ZAP-X SRS were fatigue (62.5%), headache (35.5%), gait disturbance (23.5%), hypersomnia (20%), alopecia (19%), dizziness (18.5%), radiation dermatitis (16%), nausea (7%), tinnitus (0.5%), and visual disturbance (0.5%) (Figure 5). The two most common side effects observed were fatigue and headache. Most side effects were mild or moderate and self-limited, resolving approximately within two weeks after treatment. Overall, 28.5% of patients reported no side effects.

Clinical outcomes

**Figure 6:**
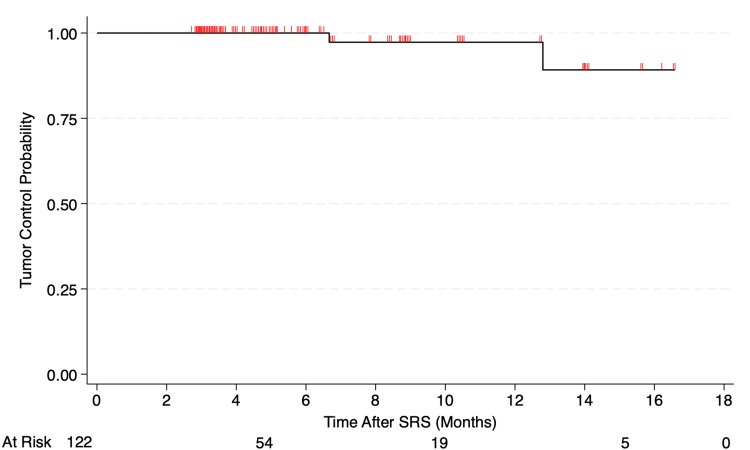
BM Kaplan-Meier tumor control curve for 40 patients (122 targets) The Kaplan-Meier local tumor control was 97% at 12 months and 89% at 16 months BM: brain metastases; SRS: stereotactic radiosurgery

For 40 BM patients consisting of 122 targets, the median follow-up was four months (IQR: p25 = 3, p75 = 8 months). Follow-up was scheduled at one month post-treatment, followed by three-month intervals, which consisted of the neurosurgeon's and radiation oncologist's direct evaluation and follow-up MRI. Complete response was defined as no visible target lesion, partial response ≥30% decrease in longest distance, stable <30% decrease in longest distance, and progressive disease ≥20% or 2.5 mm increase in longest distance [25-26]. Of note, 75 targets (61.5%) were classified as complete response, 30 (24.6%) as partial response, 15 (12.3%) as stable, and two (1.6%) as progressive disease. The Kaplan-Meier local tumor control was 97% at 12 months and 89% at 16 months. The BM Kaplan-Meier local tumor control curve is shown in Figure 6.

**Figure 7:**
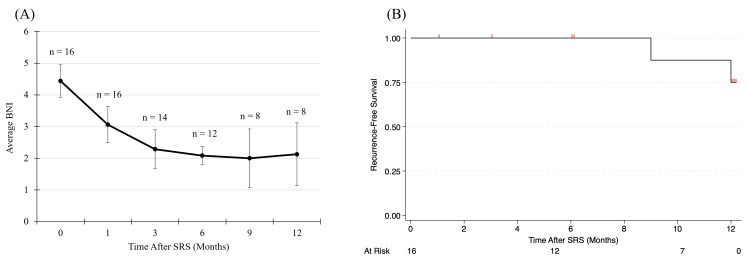
(A) Average BNI versus time after ZAP-X TN SRS. (B) TN Kaplan-Meier recurrence-free survival curve for 16 patients The recurrence-free survival was 75% at 12 months (25% recurrence rate) BNI: Barrow Neurological Institute pain scale classification; SRS: stereotactic radiosurgery

For 16 TN patients, the median follow-up was 7.5 months (IQR: 5, 12 months). Follow-up was scheduled at one month post-treatment, followed by three-month intervals, which consisted of the neurosurgeon's and radiation oncologist's direct evaluation, Modified Barrow Neurological Institute (BNI) pain scale classification [27], and follow-up MRI. A positive outcome was defined as achieving a BNI score lower than the presentation value, while recurrence was defined as an increase in the BNI score relative to the last follow-up assessment. Follow-up BNI was provided for 16 patients at one month, 14 patients at three months, 12 patients at six months, eight patients at nine months, and eight patients at 12 months. One-, three-, and six-month follow-ups demonstrated positive patient outcomes, with all patients experiencing decreased pain intensity and relief at one month. Three patients had a complete response to treatment (BNI = I, no pain, no medication): one patient at three months, two patients at nine months. The average BNI before SRS was 4.4 ± 0.5 for 16 patients, and the average BNI 12 months after SRS was 2 ± 1 for eight patients (Figure 7A). The Kaplan-Meier recurrence-free survival was 75% at 12 months (25% rate). At nine and 12 months, two patients experienced pain recurrence. The TN Kaplan-Meier recurrence-free survival curve is shown in Figure 7B.

## Discussion

The purpose of this study was to evaluate the clinical performance and initial implementation outcomes of the ZAP-X SRS system through a prospective analysis of the first 200 patients treated at JSUMC. As one of the earliest and most comprehensive clinical experiences with this novel gyroscopic, self-shielded SRS platform, this study aimed to assess treatment efficiency, patient satisfaction, and technical accuracy across a wide spectrum of central nervous system pathologies. The findings demonstrate that the ZAP-X system offers high dosimetric precision, treatment capability, and patient satisfaction, supporting its role as a viable and effective alternative to traditional SRS systems such as GK and CK. This work provides important real-world validation of the system's potential in clinical settings and lays the groundwork for further exploration and expansion of its applications.

Dosimetric indices and performance

The plan quality indices calculated in this study are similar to those reported in the literature for ZAP-X. In the first large prospective study involving the ZAP-X platform, Ehret et al. reported an average CI of 1.26 ± 0.20, PCI of 0.78 ± 0.16, GI of 3.20 ± 0.44, and HI of 1.69 ± 0.24 across 100 patients [5]. Hendricks et al. reported an average CI of 1.54 ± 0.47 across 59 patients for ZAP-X SRS [6]. Muacevic et al. reported an average PCI of 0.79, GI of 3.04, and HI of 1.89 across 41 patients for ZAP-X SRS [7]. Conroy et al. reported an average CI of 1.43 (range: 1.21 - 1.86), GI of 3.16 (2.49 - 4.88), and HI of 1.52 (1.14 - 2.00) [8]. Paddick et al. reported an average PCI of 0.830 and GI of 2.74 for benign lesions and an average PCI of 0.700 and GI of 3.83 for metastases across seven patients [28].

Patient satisfaction

One hundred patients responded to the post-treatment questionnaire. Most patients rated their ease of scheduling highly. Staff and facility ratings were high, and patients reported a high degree of satisfaction with both staff helpfulness and facility cleanliness. Physician communication was also rated highly. Patients provided mixed feedback regarding comfort during preoperative MRI and the ZAP-X treatment itself, with some patients mentioning that the thermoplastic mask was uncomfortable. Overall, satisfaction was high, and several patients left positive comments regarding the coordination of the care team. Nearly all respondents indicated that they would recommend JSUMC’s Radiation Oncology suite to others. These results demonstrate a high level of patient satisfaction with their ZAP-X experience.

Treatment time

Treatment times for ZAP-X SRS show considerable variation in the existing literature. Ehret et al. reported an average treatment time of 47 ± 30.6 minutes and an average setup time of 9.7 ± 10.0 minutes for ZAP-X SRS [5]. Hendricks et al. reported an average treatment time of 66.1 ± 36.2 minutes [6]. Muacevic et al. reported a median treatment time of 30 minutes (IQR: p25 = 26, p75 = 34 minutes) [7]. Conroy et al. reported a median treatment time of 24 minutes per lesion (range: 9-123 minutes) [8]. Gevaert et al. and Han et al. documented average treatment times of 68.1 ± 27.5 minutes for GK, 28.4 ± 8.1 minutes for CK, 16.8 ± 2.2 minutes for Novalis-Tx DCA, 21.7 ± 3.4 minutes for Novalis-Tx DMLC-IMRT, and 42 ± 16 minutes for Helical Tomotherapy [29-30]. Reducing setup time could be achieved by streamlining patient alignment, partly through the use of the Efficast head and shoulder mask with two shims. The vendor has indicated that a gyroscopic correction update for the ZAP-X system will be implemented, which may further help decrease setup time.

**Figure 8:**
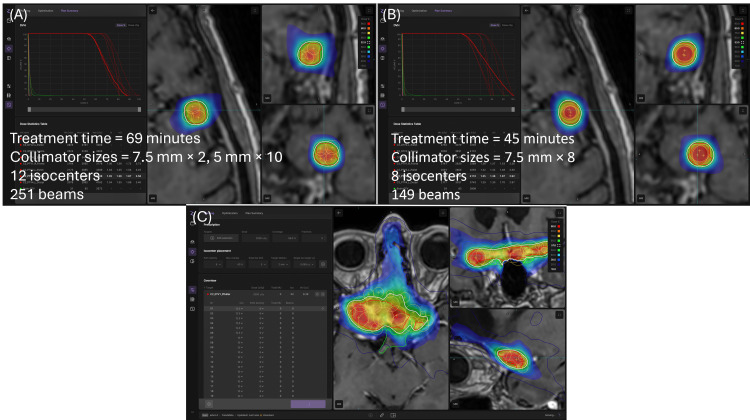
Equivalent plans on a patient where (A) one utilizes smaller collimator sizes and (B) one utilizes larger, where the larger plan has a significantly shorter treatment time. (C) Utilizing larger field sizes may not always be achievable with highly irregular shaped targets

The gyroscopic correction will use the gantry to make automatic corrections for rotational offsets (pitch, roll, and yaw) to enhance precision upon patient misalignment. The second greatest factor contributing to the treatment time was the gantry time. Currently, the ZAP-X utilizes static beams. Switching to arc-based delivery is expected to significantly reduce gantry time. According to the vendor, this upgrade is planned for implementation in the near future. The constraint for the calculated treatment time of a ZAP-X SRS plan at JSUMC is generally ≤60 minutes. To help reduce ZAP-X treatment time, the treatment planner can utilize larger collimator sizes. In our clinic, we have observed cases where two equivalent plans for the same patient (one using larger collimators and one using smaller) show that the plan with larger collimators achieves a substantially shorter treatment time, as illustrated in Figures 8A-8B. However, planners must be mindful that larger field sizes may not always be feasible for highly irregularly shaped targets, as demonstrated in Figure 8C.

Clinical outcomes

The clinical results in this study for BM and TN ZAP-X SRS are in line with those reported in the literature for SRS. In a pooled analysis of manuscripts evaluating tumor control probability for BM SRS, Redmond et al. reported that for tumors ≤20 mm, single-fraction doses of 18 and 24 Gy corresponded with >85% and 95% one-year local control rates, respectively. For tumors of 21-30 mm, an 18 Gy single-fraction dose was associated with 75% local control. For tumors of 31-40 mm, a 15 Gy single-fraction dose yielded ~69% local control. For three- to five-fraction SRS using total doses ranging from 27 to 35 Gy, one-year local control of 80% has been reported for tumors 21-40 mm in diameter. [31]. In a systematic review of TN SRS, Tuleasca et al. reported a median recurrence rate of 23% for GK, 27.2% for CK, and 29% for Linac-based [32].

Mixed fractionation optimization

To reduce ZAP-X treatment planning time for mixed fraction schemes, a method was developed utilizing one optimization run for multiple targets [33]. To accommodate mixed fractionation schemes for ZAP-X SRS, targets are first optimized in a single plan (Plan_A) using a single fractionation to their prescribed doses. After optimization, isocenters with new beams/MUs on them are split into groups with their associated targets to make individual plans (Plan_A_PTV1, Plan_A_PTV2, etc.) with proper fractionation. Each plan is then only re-prescribed to any isodose level to scale the dose to achieve 99% coverage on the respective targets. After individual plans achieve 99% dose coverage, plan sums for individual plans are compared on clinical objectives using Timmerman dose constraints. A study reporting and analyzing this ZAP-X mixed fraction optimization method is planned for a separate report.

Patient clearance

**Figure 9:**
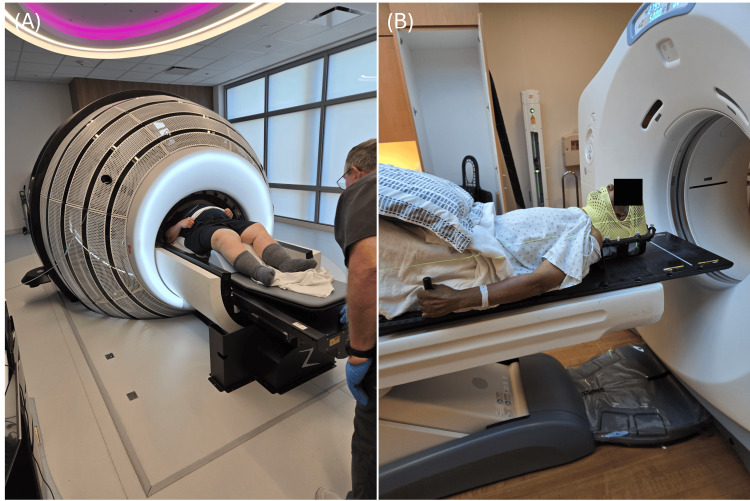
(A) Largest patient treated with ZAP-X SRS at JSUMC (weighing 300 lbs). (B) Patient requiring elevation with mold care due to difficulty breathing while lying down flat on the treatment table JSUMC: Jersey Shore University Medical Center

When planning the treatment, the dimensions of the ZAP-X relative to the patient must be considered and checked regarding larger-sized patients or patients who require accommodations during treatment. The table to shell entrance height is 45.72 cm, the shell entrance width is 81.28 cm, the table railing width is 50.8 cm, the treatment range from table to nose is 27 cm, and the table weight limit currently is 460 lbs. At JSUMC, the largest patient treated with ZAP-X SRS weighed 300 lbs (Figure 9). One patient required elevation using mold care due to difficulty breathing while lying flat.

Limitations

As this study was conducted at a single center, the findings may have limited generalizability. Outcomes could differ at other institutions with different personnel or patient populations and varying proportions of pathologies. In the future, we hope our data can contribute to a larger, multi-institutional study on ZAP-X technology. Patient satisfaction and mask evaluations may be subject to selection bias. Additionally, because the technology was recently implemented at JSUMC, follow-up time was limited. Future analyses will aim to evaluate prospective outcomes related to tumor control, symptomatic improvement, and other patient care metrics following SRS. Further studies will also include a control group, such as GK, CK, or Linac-based systems, to directly compare the efficacy of ZAP-X with other widely used SRS modalities. The results presented in this study pertain to system version DP-1010.

## Conclusions

Our experience utilizing the ZAP-X radiosurgery platform has been extremely positive. Since its implementation at JSUMC, treatment of both benign and malignant lesions alike has been effective and without complications. Across the board, our Neurosurgery and Radiation Oncology Departments effectively synergized to deliver a streamlined treatment planning workflow, which contributed to excellent patient satisfaction. We look forward to exploring future directions with this novel radiotherapy option and expanding the scope of its utilization to other pathologies of the central nervous system and beyond. As the body of literature on ZAP-X continues to grow, we anticipate that its benefits will draw wider adoption by physicians worldwide. In this context, our findings can help guide the refinement and optimization of the ZAP-X system for a broad range of clinical indications.
